# Evaluation of integrative oncology modalities for symptom management: a MASCC/SIO global survey

**DOI:** 10.1186/s12906-025-05157-6

**Published:** 2025-11-03

**Authors:** Alexandre Chan, Reem Nasr, Daniela Arcos, Dalia Kagramanov, Chioma Asuzu, Ting Bao, Yin Ting Cheung, Jung Hye Kwon, Judith Lacey, Richard T. Lee, Maryam Lustberg, Beatrice M. Ohaeri, Santosh Rao, Enrique Soto-Perez-de-Celis, Claudia M. Witt, Ana Maria Lopez

**Affiliations:** 1 Department of Clinical Pharmacy Practice, University of California Irvine, 802 W Peltason Drive, Irvine, CA 92697-4625 USA; 2 Department of Guidance and Counselling, University of Ibadan, Ibadan, Nigeria; 3 Zakim Center for Integrative Therapies and Healthy Living, Dana-Farber Cancer Institute, Boston, USA; 4 School of Pharmacy, Faculty of Medicine, The Chinese University of Hong Kong, Hong Kong SAR Shatin, China; 5 Division of Hemato-oncology, Chung-Ang University College of Medicine, Gyeonggi-do, South Korea; 6 Department of Supportive Care and Integrative Oncology, Chris O’Brien Lifehouse Comprehensive Cancer Centre, NSW Sydney, Australia; 7 Cherng Family Center for Integrative Oncology, City of Hope Comprehensive Cancer Center, Duarte, USA; 8 Breast Cancer Center, Smilow Cancer Hospital, Yale University, New Haven, USA; 9 College of Medicine, University of Ibadan, Ibadan, Nigeria; 10 University Hospitals Seidman Cancer Center, Cleveland, USA; 11 Department of Geriatrics, Instituto Nacional de Ciencias Médicas y Nutrición Salvador Zubiran, Ciudad de Mexico, Mexico; 12 UC Health Diane O’Connor Thompson Breast Center, Auschutz Medical Campus, University of Colorado, Aurora, USA; 13 Institute for Complementary and Integrative Medicine, University Hospital Zurich, Zürich, Switzerland; 14 Sidney Kimmel Comprehensive Cancer Center, Thomas Jefferson University, Philadelphia, USA

**Keywords:** Integrative oncology, Cancer supportive care, Oncology, Cancer, Global perspectives

## Abstract

**Background:**

With growing evidence pointing towards the potential of integrative oncology modalities (IOM) in addressing cancer and cancer-treatment related symptoms, research on IOM utilization and implementation is warranted. This study examines global stakeholder perspectives on integrative oncology (IO) utilization for supportive cancer care.

**Methods:**

Members of the Multinational Association of Supportive Care in Cancer (MASCC) and the Society for Integrative Oncology (SIO) completed a survey on the utilization of IOM for supportive cancer care. Descriptive statistics were used to assess demographic data, IOM usage patterns, IOM education, and financial considerations for utilizing IOM.

**Results:**

Among 344 participants representing eight geographical regions, 70% reported having utilized or recommended IOM and 79% perceived IOM to be underutilized in cancer supportive care. Acupuncture (48%), exercise classes (39%), nutrition (38%), breathing/yoga (38%) and personalized exercise (38%) were among the most utilized IOM across regions. Relatedly, the symptoms for which IOM were most recommended for persons with a diagnosis of cancer in active treatment [AT] or completed treatment [CT] respectively were emotional (AT 23%; CT 26%), pain (AT 22%; CT 20%), gastrointestinal (AT 21%; CT 12%) and fatigue (AT 15%; CT 16%). The perceived availability of integrative medicine training was highest in North America (69%). Across regions, self-pay (20%-67%), private insurance (0%-26%) and government insurance (7%-40%) were the most common forms of payment for IOM. The IOM most recommended in high-income countries (acupuncture, exercise, massage, individual exercise) varied from the IOM most recommended in low-middle income countries (nutrition counseling, exercise classes, breathing, acupuncture).

**Conclusion:**

This study provides valuable insights into global utilization patterns of IOM in supportive cancer care, highlighting that while most respondents have utilized IOM, there is a perceived underutilization overall. Our results show significant regional differences in the availability of integrative oncology education and hint to financial barriers impacting IOM use. Further research is necessary to explore these aspects and inform strategies for supporting IOM implementation efforts.

**Supplementary Information:**

The online version contains supplementary material available at 10.1186/s12906-025-05157-6.

## Introduction

Integrative oncology (IO) is a “patient-centered, evidence-informed field of cancer care that utilizes mind-body practices, natural products, and lifestyle modifications from varying global traditions alongside conventional cancer treatments”[1–3]. Integrative oncology aims to optimize health, quality of life, and clinical outcomes across the cancer care continuum and to empower people to prevent cancer and become active participants before, during, and beyond cancer treatment [3]^”^. The National Center for Complementary and Integrative Health (NCCIH) highlights that integrative oncology modalities (IOM) are meant to act as a complement to traditional treatment avenues and not to replace conventional cancer therapies - enhancing a comprehensive approach to care for those with cancer [1, 4].

Within the cancer context, supportive care is defined by the Multinational Association of Supportive Care in Cancer (MASCC), a leading global organization of healthcare professionals and researchers in supportive cancer care, as “the prevention and management of the adverse effects of cancer and its treatment”. Recent studies have shown that supportive care in cancer is an “indispensable component,” as it aims to improve the quality of life of the patient throughout the entire treatment process by using a coordinated holistic approach that ensures the patient is receiving the maximum benefits from treatment⁷. Due to the growing body of evidence highlighting the efficacy of IOM for cancer-related supportive care, the use of complementary and integrative medicine alongside conventional therapies has greatly increased [1]. Moreover, integrative therapies have also been added to the American Society for Clinical Oncology’s (ASCO) guidelines, with ASCO endorsing the Society for Integrative Oncology’s (SIO) Clinical Practice Guidelines and working with SIO to develop evidence-informed guidelines for common cancer-related symptoms, such as pain, anxiety, and fatigue. Similarly, within its Supportive and Palliative Care Clinical Practice Guidelines, the European Society for Medical Oncology (ESMO) recommends evidence-based IOM such as mindfulness-based interventions and exercise for support of symptoms such as anxiety, depression and pain [5]. The National Comprehensive Cancer Network (NCCN), which collaborates with various oncology organizations worldwide, has also included integrative medicine guidelines for reference by practitioners and other stakeholders, exemplifying a growing recognition of integrative therapies within oncology practice guidelines [1, 6–9].

Some IOM can be practiced independently or with minimal introductory guidance (through modalities such as breath work, meditation, and exercise), which can enrich a patient’s sense of autonomy and engagement in managing their own care – empowering them to take an active role in their health [10]. Mindfulness based interventions, for example, have been found to reduce pain, stress, depression and anxiety in patients with cancer [11], improving their quality of life (QoL) [12]. Similarly, a significant body of research suggests that exercise, particularly with a personalized exercise program, can decrease the severity of various cancer and treatment-related symptoms, improving psychological health, aerobic fitness, and QoL [13]. Relatedly, studies have reported promising effects of yoga in improving physical (e.g., fatigue, pain, physical discomfort) and psychological or mental symptoms (e.g., anxiety, depression, cognitive function) [14, 15]. IOM administered by practitioners and working with IOM such as acupuncture, massage, reflexology, reiki, and guided imagery, have also been shown to alleviate pain, neuropathy, nausea and vomiting, fatigue, anxiety, and depression, among other ailments commonly experienced by persons with cancer [11, 14–16].

Although the current literature has mostly focused on understanding the benefits of IOM in addressing cancer-related symptoms, there is a growing interest in exploring the effective implementation of evidence-based IOM into existing cancer care models (i.e. within cancer centers and specialized clinics). Some research has shown that comprehensive cancer centers are increasingly working to incorporate IOM services to meet the widespread interest for IOM among patients [17]. However, there is limited data to show the ways in which these services are provided, how they are accepted by stakeholders, and the key facilitators and barriers to integrate these modalities effectively. Few studies have focused on assessing utilization and implementation factors at a global level. This information is valuable, as it can provide insight into how factors that differ across countries, such as cultural elements or differing health systems, may impact the use of IOM – guiding future areas of focus.

As a result, the primary objective of this survey was not merely to collect opinions, but to use expert perspectives to critically evaluate the global utilization, accessibility, and integration of Integrative Oncology Modalities (IOM) in cancer supportive care. By surveying members of the Multinational Association of Supportive Care in Cancer (MASCC) and the Society of Integrative Oncology (SIO), the study aimed to identify key barriers, regional disparities, and implementation challenges. This first manuscript is focused to describe the global use of IOM for symptom management, the education landscape related to IOM in supportive care, and global financial landscape of IOM. These findings were intended to inform future policy, education, and research efforts aimed at improving the global integration of IOM into oncology care.

This study will also provide a better understanding of global stakeholder perspectives and guide future research in the field of integrative oncology.

## Methods

### Survey design

A cross-sectional, twenty-question survey was developed specifically for this study by a panel of 13 interprofessional MASCC/SIO members with expertise in integrative oncology and supportive cancer care. The survey was iteratively designed through collaborative meetings to ensure alignment with the study’s objectives, which focused on assessing global utilization, accessibility, and implementation challenges of Integrative Oncology Modalities (IOM) in cancer supportive care. The final survey included a mix of multiple-choice, Likert scale, and open-ended questions, and was pilot-tested by members not involved in the survey design to ensure clarity and relevance. Furthermore, an interdisciplinary team of thirteen MASCC and/or SIO members reviewed the survey questions before they were assessed by the MASCC Executive Committee for prior to circulation by task force members and by the MASCC board of directors for face and content validity. Other psychometric testings were not conducted.

All active members of the Multinational Association for Supportive Care in Cancer (MASCC) and/or the Society for Integrative Oncology (SIO) were eligible to complete the survey and received an anonymous email invitation between June and August 2023, with two follow-up reminders. MASCC is a global society of healthcare professionals and researchers dedicated to cancer supportive care, while SIO is a multidisciplinary professional society focused on evidence-based integrative oncology. The study received approval from the University of California, Irvine Institutional Review Board (Protocol #3392), with a waiver of informed consent. Data were analyzed using IBM SPSS Statistics Version 29.0.0.0. (The full survey is available in the Supplementary Material Files.)

#### Inclusion/exclusion criteria

All active MASCC and SIO members in 2023 were eligible for participation and received an invitation to the survey via email. At the time of dissemination, MASCC had 2,129 verified members across 70 countries, comprising a broad spectrum of healthcare professionals including physicians, nurses, pharmacists, dentists, dietitians, physiotherapists, psychologists, and others who contribute to supportive care in oncology. Membership requires professional verification upon joining the organization. SIO had 585 members at the time, representing a multidisciplinary network of oncologists, nurses, psychologists, social workers, nutritionists, complementary therapy practitioners, naturopathic doctors, herbalists, acupuncturists, yoga therapists, massage therapists, and other professionals working within integrative oncology. These members span academic medical centers, private practices, industry, and community-based settings.

To enhance the relevance of responses and ensure alignment with the study’s objectives, the survey introduction clearly stated that the purpose was to understand global practices related to the use of integrative oncology modalities (IOMs) specifically within cancer supportive care. While no formal exclusion criteria were enforced during dissemination, respondents were asked to complete the survey only if their professional activities involved or were related to the care of individuals with cancer. Although the survey did not include mandatory screening questions (e.g., number of cancer patients seen per year), several items within the questionnaire explored the respondent’s profession, practice setting, region, and scope of involvement with IOMs and oncology care—allowing for subsequent stratification of responses and exclusion of clearly irrelevant data, if necessary, during analysis.

### Data collection & survey information

The 22-question survey was built in Qualtrics (Irvine, CA), a secure online data capture platform, and took participants approximately 15 to 20 min to complete. Items were not randomized or alternated, adaptive questioning was not utilized, and completeness checks and review of answers were not included in the survey. In this manuscript, we are reporting information on (1) participant demographic characteristics, (2) utilization and recommendation of IOM for patient symptoms, (3) perceived presence of supportive/palliative care and integrative medicine training, and (4) forms of payment for IOM services.

The survey was divided into four sections. The first section included nine items inquiring about the demographic characteristics of the participant (i.e. age, country of practice, professional role(s), and time worked in professional role(s)). Questions were asked in a ‘fill-in-the-blank’ or ‘select which applies’ manner. The second section, which included seven items inquired about the presence and accessibility of supportive/palliative care and integrative medicine training in the participant’s country of practice, the conduction of integrative oncology assessments/consultations, and how integrative oncology services are paid for. Questions were similarly presented in a ‘fill-in-the-blank’ or ‘select which applies’ format.

The third section presented a table that would assess the participant’s utilization and recommendation of IOMs to manage patient symptoms. A table outlining several IOM modalities was provided for participants to indicate usage (‘yes/no’) and frequency of use (‘sometimes, often or always’). Lastly, the fourth section assessed the top symptoms that providers would recommend the utilization of IOMs for patients undergoing primary treatment and for survivors who have completed primary treatment. Participants were given space to type in their top 5 recommendations for each, as well as indicate whether they believe IO is underutilized in supportive care.

The survey platform was open and not password-protected. Invitations to participate in the survey were disseminated via an initial email link sent to MASCC and SIO members in June 2023, followed by two email reminders, remaining open from June to August 2023. No incentives were provided for answering the survey.

### Statistical analysis

All data were analyzed using IBM SPSS Statistics Version 29.0.0.0. Descriptive statistics were used to assess demographical data and varied IOM usage patterns. Categorical data are presented as counts and percentages, while continuous data on demographic variables are presented as means and standard deviation.

## Results

### Demographic characteristics

A total of 2,714 emails were sent out for recruitment across both organizations (MASCC and SIO) and 431 responses were collected (response rate of 15.9%). Of the responses collected, 344 (79.8%) were completed beyond the initial demographic information section and, as such, were included in the analysis. Table 1 summarizes the demographic characteristics of our sample. Majority of respondents were female (63.4%), and members of MASCC (68.6%), SIO (14.5%), or both (8.4%). The mean (SD) age was 50.7 (12.8) and the mean (SD) number of years worked was 17.2 (11.0), with most participants working in the public (37.5%) and academic (35.8%) sectors. Overall, most respondents worked in high (83.1%) or middle-upper (8.4%) income countries; reported residence in North America (34.3%), East Asia and the Pacific (30.8%), or Europe (19.8%). The most commonly reported primary professions were Physicians (44.6%) and Clinical Science Researchers (11.9%).

**Table 1:** Demographic characteristics (n = 344)

	*N*	Percent	Mean	SD
Age			50.66	12.77
Gender				
Female	218	63.4		
Male	120	34.9		
Non-binary/Other	6	1.7		
Member of				
MASCC	236	68.6		
SIO	50	14.5		
Both	29	8.4		
None	29	8.4		
Years worked			17.16	11.02
Work sector				
Academic	123	35.8		
Mixed public/private	39	11.3		
Public	129	37.5		
Private	53	15.4		
Region* (work/practice in)				
Central Asia	4	1.2		
East Asia & Pacific	106	30.8		
Europe	68	19.8		
Latin America and Caribbean	12	3.5		
Middle East and North Africa	10	2.9		
North America	118	34.3		
South Asia	18	5.2		
Sub-Saharan Africa	5	1.5		
Unspecified	3	0.9		
Country’s income level*				
High	286	83.1		
Middle-Upper	29	8.4		
Lower-Middle	25	7.3		
Low	1	0.3		
Unspecified	3	0.9		
Primary Profession				
Physician (working/certified in integrative health)	85	24.7		
Physician (not working/certified in integrative health)	73	21.2		
Clinical Science Researcher	47	13.7		
Nurse	27	7.8		
Dentist/Oral Surgeon	22	6.4		
Advance Practice Provider	14	4.1		
Patient Advocate	12	3.5		
Pharmacist	11	3.2		
Acupuncturist	9	2.6		
Physiotherapist	6	1.7		
Administrative Position	5	1.5		
Other**	33	9.6		
Secondary Profession				
Not applicable/No Secondary Profession	97	28.2		
Clinical Science Researcher	61	17.7		
Physician (working/certified in IH)	35	10.2		
Nurse	27	7.8		
Physician (not working/certified in IH)	20	5.8		
Patient Advocate	12	3.5		
Advance Practice Provider	9	2.6		
Psycho-Oncologist	8	2.3		
Trainee/Student	8	2.3		
Basic Science Researcher	7	2.0		
Pharmacist	7	2.0		
Acupuncturist	6	1.7		
Health/Lifestyle Coach/Counselor	5	1.5		
Traditional Oriental Medicine Practitioner	5	1.5		
Other**	37	10.8		

*Countries’ region and income level classied according to the World Bank (12)

**Other (n ≤ 4 per category; Entries include: Basic Science Researcher, Psycho-oncologist, Nutritionist, Psychologist, Social Worker, Traditional Oriental Medicine Practitioner, Mind-body Therapist, Accredited Exercise Physiologist, Ayurvedic Medicine Practitioner, Dental Hygienist, Fitness/Exercise Instructor, Medical Herbalist, Mindfulness/Meditation Teacher, Naturopath, Radiation Therapist, Shiatsu Therapist, Side Effects Expert, and Yoga therapist)

### Utilization and recommendation of IOM to manage patient symptoms

**Fig. 1:**
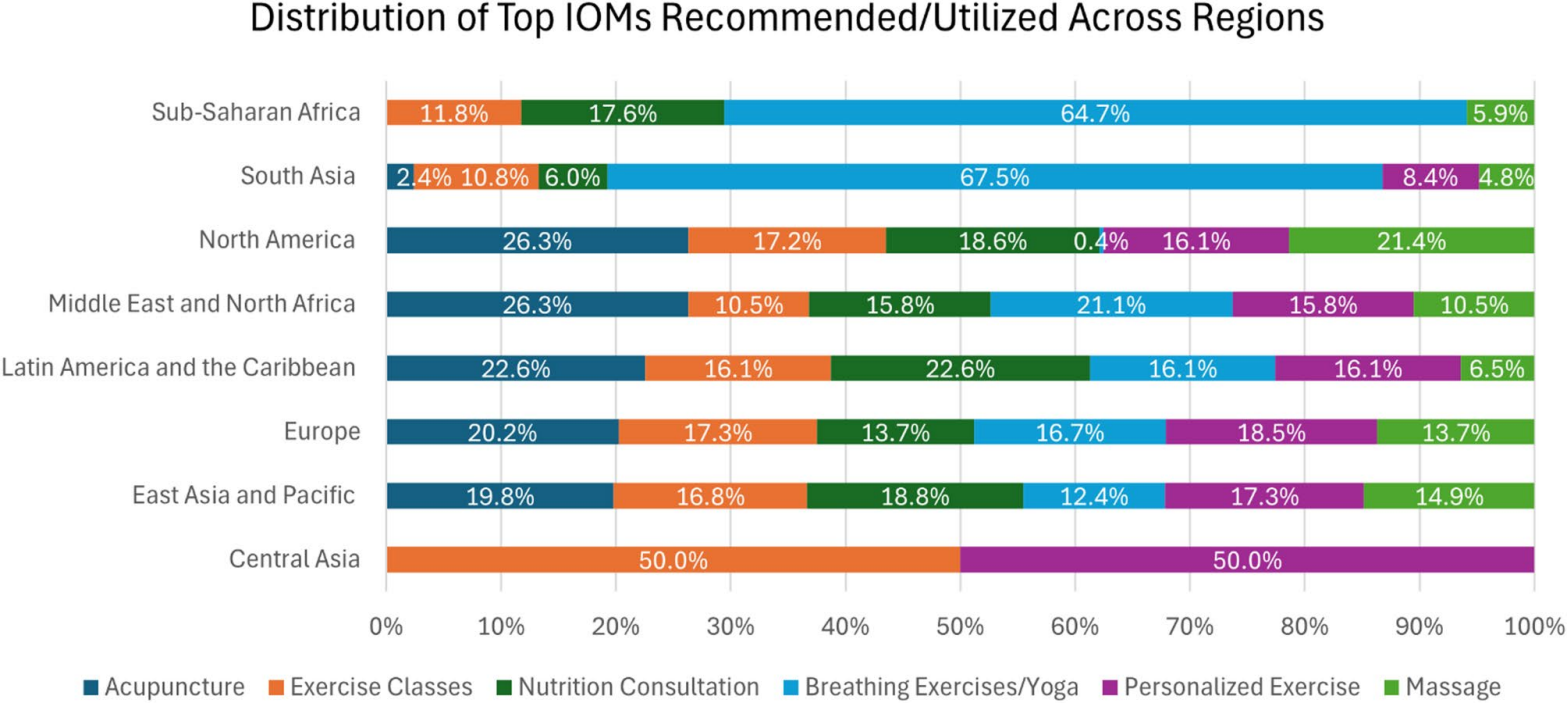
Distribution of top IOMs recommended/utilized across Regions-% based on total responses per region

Across the overall sample, 78.7% noted that they felt IOM were underutilized and 69.8% reported using or recommending at least one IOM to their patients for supportive care issues within their career (see Supplementary Table 1 for breakdown of IOM utilized or recommended). The six IOM most often recommended were acupuncture/acupressure (47.7%), exercise classes (38.7%), nutrition consultation (38.4%), breathing exercises/yoga (37.8%), personalized exercise (37.5%), and massage (36.0%) (Fig. 1: Distribution of Top IOMs Recommended/Utilized Across Regions-% based on total responses per region).

When participants were probed further, participants were asked to report on IOM they utilize or recommend to manage their patients symptoms on an annual, monthly, or weekly basis (Supplementary Table 2). The top three IOM’s recommended annually were acupuncture/acupressure (15.1%), art therapy (9.3%), and aromatherapy (5.8%). Monthly, the top three IOM included massage (12.2%), nutrition consultation (11.9%), and peer support (11.3%). On a weekly basis, the top three IOM recommended were personalized exercise (21.2%), exercise classes (18.9%), breathing and exercises/yoga (17.2%).

In addition, respondents provided information on the symptoms for which they recommend the use of IOM. These symptoms were organized into ten categories: Emotional, pain, gastrointestinal, fatigue, sleep, hormonal, mobility, dyspnea, cognitive, and other (see Supplementary Table 3 for term definitions). The top symptom categories in patients undergoing active primary treatment for which IOM were recommended were emotional (22.7%), pain (21.5%), gastrointestinal (20.7%), and fatigue (15.1%).

**Fig. 2:**
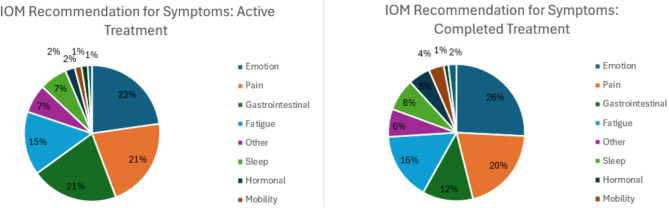
IOM Recommended - symptom categories (active and completed treatment

Similarly, for patients who had completed their primary treatment, the top symptom categories for which IOM were recommended were emotional (25.8%), pain (20.3%), fatigue (15.9%), and gastrointestinal (12%) (Fig. 2: IOM Recommended - Symptom Categories (Active and Completed Treatment)).

Supplementary Figs. 1 and 2 illustrate the distribution of the symptoms for which IOM were recommended by region for patients undergoing active primary treatment and for patients who have completed primary treatment, respectively.

### Education and training on integrative oncology

Participants indicated if they had received formal supportive care/palliative care (SCPC) or integrative medicine (IM) training. The majority reported not having received SCPC training (63.1%) and not having received IM training (80.8%). Of those who did report having received SCPC training (36.9%), most received the training in North America (29.1%), East Asia and Pacific (27.6%), or Europe (18.1%). Similarly, of those who reported having received formal IM training (19.2%), most received training in North America (42.4%) followed by East Asia and Pacific (15.2%).

**Fig. 3:**
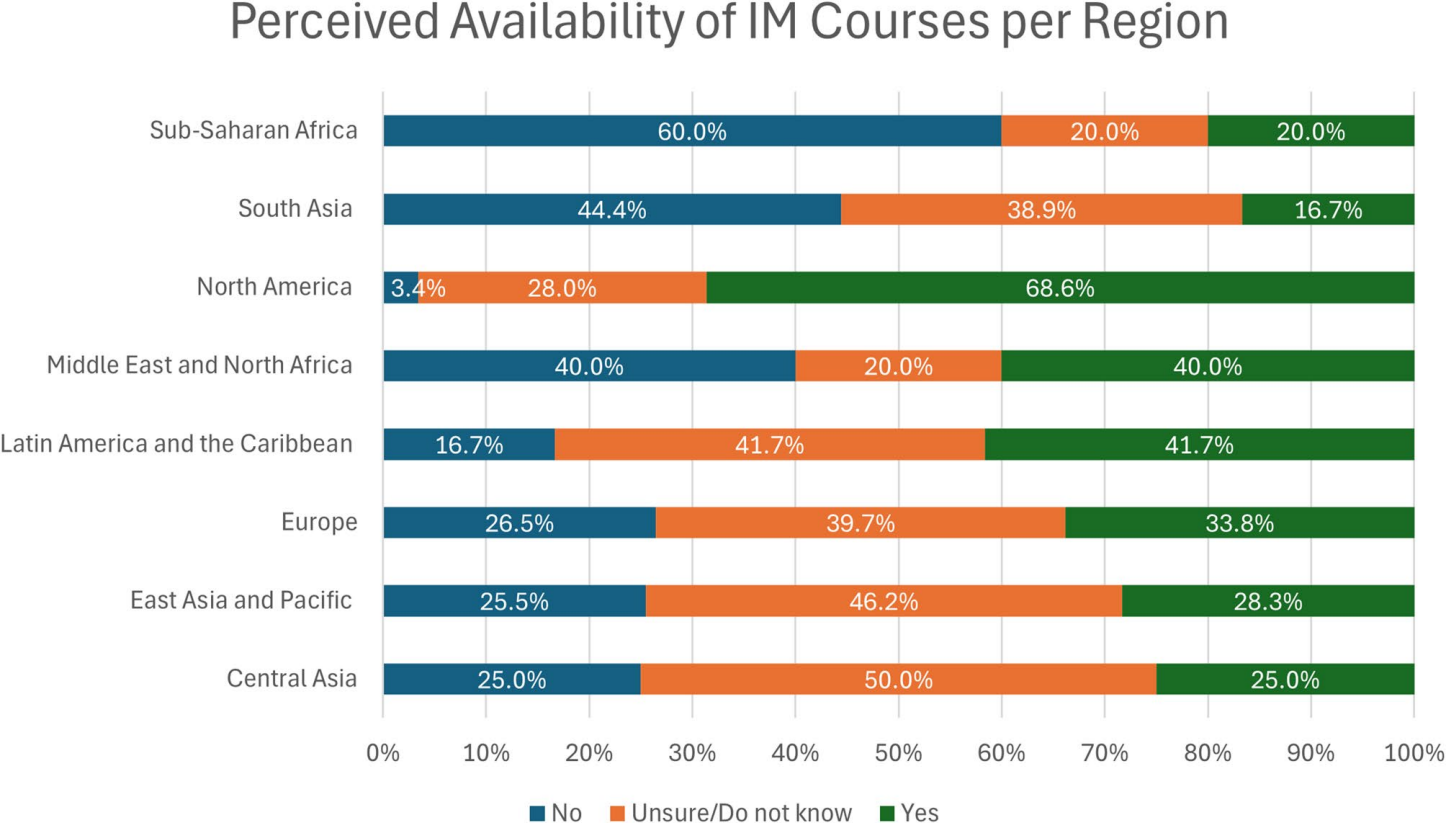
Perceived availability of IM courses per Region- % based on total responses per region

Respondents also indicated the perceived availability of IM courses in their region (Fig. 3: Perceived Availability of IM Courses per Region- % based on total responses per region).

North America (68.7%), followed by Latin America and the Caribbean (41.7%), were the regions with the highest percentage of respondents indicating the availability of IM courses. In contrast, South Asia (16.67%) and Sub-Saharan Africa (20%) were the regions with the lowest percentage of respondents indicating perceived availability of IM courses.

### Global financial landscape of IOM

**Fig. 4:**
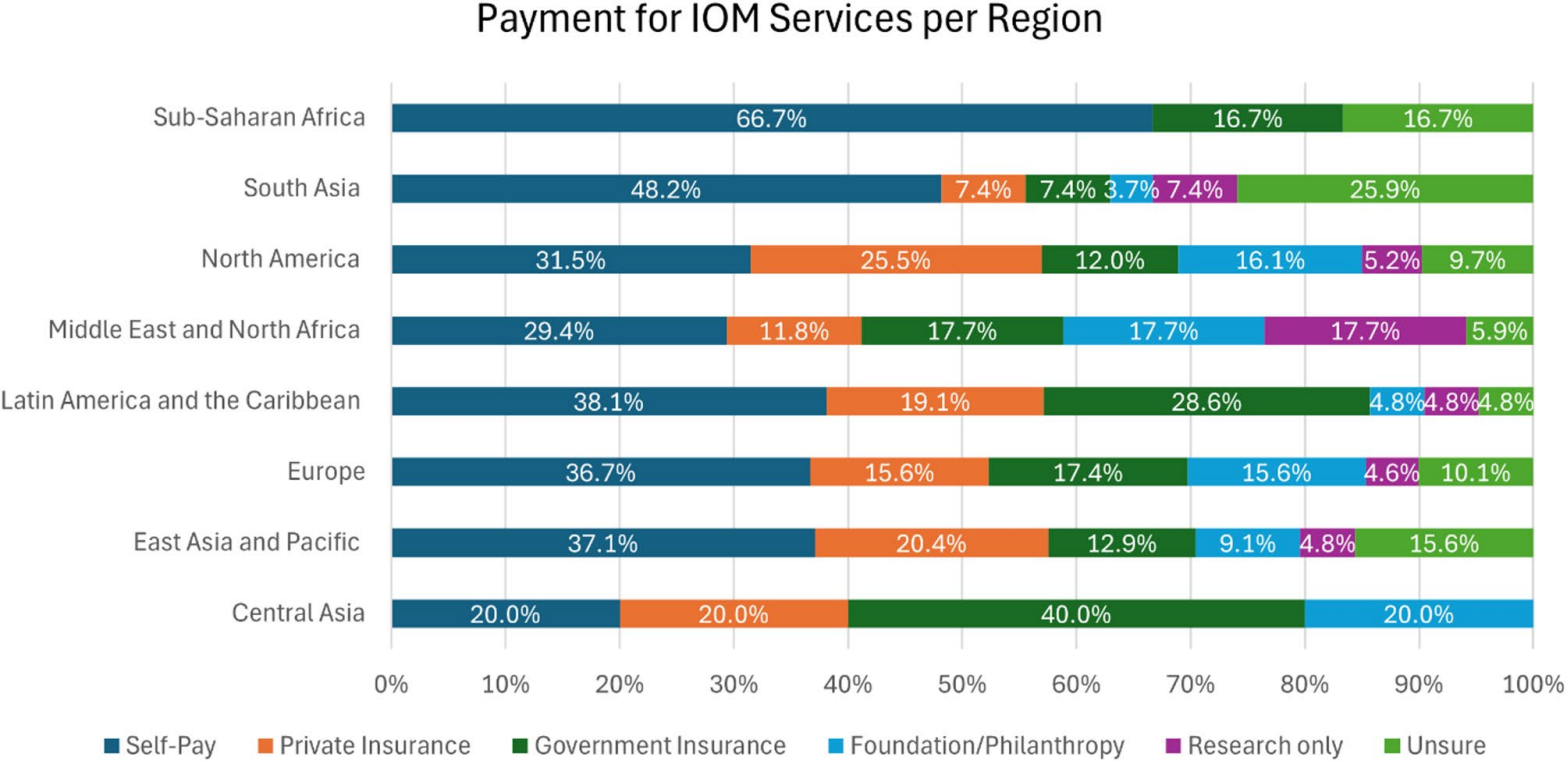
Payment for IOM services per Region-% based on total responses per region

Participants reported how IOM is paid for in their country of practice (illustrated in Fig. 4: Payment for IOM Services per Region-% based on total responses per region). In most regions, self-pay (20%−66.5%), private insurance (0%−25.5%), and government insurance (7.4%−40%) were the most commonly mentioned forms of payment for IOM services (accounting for 63%−85.7% of all responses). In contrast, foundation/philanthropy (0%−20%), research (0%−17.6%), or ‘unsure’ (0%−25.9%) accounted for less than 50% of the responses across regions (16.7%−41.2%).

When comparing IOM utilization based on countries’ income level, among participants from high income countries (HIC) (83.1%), 70.6% report having recommended at least one IOM. In contrast, among respondents from low-middle income countries (LMIC) (15.7%), 65.5% report having recommended at least one IOM. Relatedly, the most commonly utilized or recommended IOM in HIC were acupuncture (42.7%), exercise classes (35.7%), massage (35.5%), and individualized exercise prescription (34.6%). In comparison, among LMIC, nutrition counseling (37%), exercise classes (33.3%), breathing exercises (33.3%), and acupuncture (33.3%) were the most commonly recommended IOM. (Fig. 4: Payment for IOM Services per Region-% based on total responses per region).

## Discussion

This study investigated the availability and utilization of IOM in cancer supportive care across different geographical regions. We found that although a majority of respondents from our sample, comprised of MASCC and/or SIO members, had utilized or recommended at least one IOM for supportive care, yet they still perceived IOM to be underutilized. The IOM most utilized or recommended were acupuncture/acupressure, exercise (classes and personalized programs), nutritional guidance, breathing exercises (including yoga), and massage. Similarly, our results show that IOM are most often recommended to address emotional or psychological distress, pain, gastrointestinal symptoms, and fatigue, exemplifying the prevalence of these symptoms and their impact on QoL among persons with cancer [18]. A recent cross-sectional survey of researchers and clinicians on complimentary, alternative and integrative medicine (CAIM), also reported perceiving mind-body therapies such as yoga, meditation, breathwork to be most supportive in oncology [19]. This collective information illustrates the professional understanding that IOM may be of benefit. Existing evidence-based guidelines [6, 11, 14] that highlight using IOM to address such symptoms may reinforce use by clinicans.

Our results also provide valuable information on the availability of IO education for health professionals. While a majority of respondents had not received formal SCPC or IM training, those who had received formal education in these areas did so mostly in North America. In agreement, most respondents residing in North America indicated perceived availability of IM courses, in contrast to a lower percentage of participants from other regions perceiving availability of such courses. It is reasonable to consider that geographical limitations in IM education availability may be a factor impacting IOM utilization, as fewer health professionals would have the necessary training to deliver IM services to their patients. Relatedly, lack of available education could be linked to lack of awareness for both patients and healthcare professionals, reducing interest in and utilization of IOM in cancer supportive care [20]. As an emerging field, IO currently lacks widespread access to formal training programs such as fellowships, standardized certification pathways, and structured academic curricula in many parts of the world. This gap in advanced educational opportunities may contribute to inconsistencies in clinical practice, limited workforce capacity, and uneven implementation of IOM across healthcare systems [21]. Currently, there is web-based integrative oncology education that is developed for patient navigators; further studies should be conducted whether such curriculum can be extended to patients and healthcare professionals. Our results support findings from a previous study [22], where a sample of professionals from six countries reported that both lack of awareness and funding acted as current barriers to IOM implementation. Recognizing this need, the MASCC and the SIO have emphasized the importance of IOM education among their members. The SIO Education Committee has developed a number of free online educational modules for international IOM practitioners and oncology professionals. These modules provide education on evidence-informed IOM for common cancer-related symptoms such as pain, anxiety, depression, and fatigue, based on the SIO/ASCO guidelines [23].

The data provided in this study allow us to explore financial considerations that may impact IOM utilization. We found that a similar percentage of participants from HIC, compared to participants from LMIC, have utilized or recommended at least one IOM. The IOM most often recommended by participants from HIC (e.g., acupuncture, exercise classes, massage, personalized excercise) varied slightly from those most often recommended in LMIC (e.g., nutritional counseling, exercise classes, breathing excercises, acupuncture). Addressing financial considerations is crucial to mitigating disparities in cancer supportive care.[24] To facilitate the inclusion of integrative oncology modalities within government-funded programs and private insurance coverage, it is imperative that only interventions supported by rigorous scientific evidence are incorporated into locally adapted clinical guidelines. Furthermore, active engagement in health policy development is essential to promote equitable access to evidence-based IOM for supporting cancer patients.

While one must be cautious to draw conclusions due to a skewed representation of HIC in our sample, it is possible that financial factors play a role in the modalities utilized or recommended. IOM representing little to no cost to patients - such as breathwork or exercise classes (compared to individualized exercise) - may be more accessible and, as such, preferred among professionals in LMIC. These modalities are relatively safe to be conducted remotely or independently by the patients (with or without caregivers) once they are taught the basic techniques. Hence, patient education and patient partnership is a future direction to facilitate implementation of these low to no cost modalities to patients. Future research may also consider further exploring the relationship between costs of different modalities and utilization across countries with differing financial landscapes. Relatedly, research that further delves into health system-level factors, including payment structures (i.e. insurance), and their relationship with IOM utilization may provide valuable implementation insights.

Our results also show that, across regions, IOM are mostly paid for through self-pay or private insurance. With financial burden often representing a significant challenge to patients with cancer [6] in the United States, it is expected that IOM utilization may be impacted by financial constraints, particularly if patients are required to pay out-of-pocket. Our data hints at the importance of financial considerations in IOM use which may also be influenced by cultural beliefs, social or political factors, healthcare systems, education, public awareness and country’s income level.

With a considerable sample, our study included respondents with varied backgrounds in terms of profession, age, race, years of experience, and country of origin. Nonetheless, several limitations should be acknowledged. The limited number of participants from certain regions—such as Central Asia and Sub-Saharan Africa—restricts our ability to draw conclusions about IOM utilization in these areas and limits meaningful regional comparisons. Additionally, representation from low- and middle-income countries (LMICs) was low (less than 10%), which reduces the diversity of perspectives captured and the generalizability of findings to those contexts. Our sample also included relatively few respondents in key roles within oncology supportive care, such as patient advocates, which may have further limited the breadth of insights obtained. Moreover, important clinical factors that can influence IOM utilization, such as cancer stage and type, were not collected. IOM is more commonly offered to patients with early-stage cancers, and regions like North America—with a higher proportion of early-stage diagnoses—may therefore report greater IOM use. Similarly, IOM is more frequently used in certain cancer types, particularly breast and prostate cancer. The lack of data on these variables limits our ability to fully explore patterns of IOM utilization across populations and care contexts. This study was limited to MASCC and SIO members and the response rate was at 15.7%. Thus, results of this study may be threatened by non-response bias as the results may not represent views of all members. However, our study’s survey approach also allows us to provide an overview on the IOM that are commonly used by cancer supportive care providers and scientific experts.

## Conclusion

This study offers important insights into the global utilization patterns of IOM, education about IOM, and the income-related barriers to accessing IOM services. Further research is necessary to assess awareness and implementation on a global scale, which could help in enhancing the use of these modalities in providing supportive care for cancer patients worldwide.

## Supplementary Information

Supplementary Material 1.

Supplementary Material 2.

## Data Availability

The datasets used and/or analysed during the current study are available from the corresponding author on reasonable request.

## Abbreviations

IOM: Integrative Oncology Modalities
IO: Integrative Oncology
MASCC: Multinational Association of Supportive Care in Cancer
SIO: Society for Integrative Oncology
AT: Active Treatment
CT: Completed Treatment
NCCIH: National Center for Complementary and Integrative Health
ASCO: American Society for Clinical Oncology
ESMO: European Society for Medical Oncology
NCCN: National Comprehensive Cancer Network
QoL: Quality of Life
SCPC: Supportive Care/Palliative Care
IM: Integrative Medicine
HIC: High Income Countries
LMIC: Low-Middle Income Countries
CAIM: Complimentary, Alternative and Integrative Medicine
