# Supplementary material for: Evaluation of integrative oncology modalities for symptom management: a MASCC/SIO global survey
Source: BMC Complement Med Ther. 2025 Nov 3;25:406. doi: 10.1186/s12906-025-05157-6 (PMC12581493; doi:10.1186/s12906-025-05157-6)

**Supplementary Material**

**Supplement Table 1: IOMs Utilized or Recommended to Manage Patients Symptoms (Ranked in descending order)**

|  | **Yes (%)** | **No (%)** |
| --- | --- | --- |
| Acupuncture/Acupressure | 47.7 | 53.3 |
| Exercise Classes | 38.7 | 61.3 |
| Nutrition Consultation | 38.4 | 61.6 |
| Breathing Exercises/Yoga | 37.8 | 62.2 |
| Personalized Exercise | 37.5 | 62.5 |
| Massage (including Oncology Massage) | 36 | 64 |
| Lifestyle Change Counseling and Guidance | 33.4 | 66.6 |
| Meditation and mindfulness | 30.2 | 69.8 |
| Peer Support | 28.8 | 71.2 |
| Art Therapy | 23.3 | 76.7 |
| Health Coaching/ Supportive Counseling | 22.7 | 77.3 |
| MBSR MBCT | 20.3 | 79.7 |
| Music Therapy | 19.2 | 80.8 |
| Aromatherapy | 18.9 | 81.1 |
| Medical/Nursing integrative oncology consultation | 18.9 | 81.1 |
| Qi Gong/Tai Chi | 14.5 | 85.5 |
| Herbal Botanicals | 14 | 86 |
| Guided Imagery | 13.4 | 86.6 |
| Biofield Therapy | 10.8 | 89.2 |
| Pet Therapy | 10.5 | 89.5 |
| Hypnosis | 9.3 | 90.7 |
| Progressive muscle relaxation | 9.3 | 90.7 |
| Dance Movement Therapy | 9 | 91 |
| Manual Therapy | 7.8 | 92.2 |
| Biofeedback | 7.6 | 92.4 |
| Reflexology | 7.6 | 92.4 |
| Homeopathy | 6.1 | 93.9 |
| Light Therapy | 5.5 | 94.5 |
| Chiropractic | 5.2 | 94.8 |
| Culinary Therapy | 5.52 | 94.8 |
| Ayurveda | 4.1 | 95.9 |
| Intravenous Vitamins | 3.8 | 96.2 |
| Laughter Therapy | 3.8 | 96.2 |
| Narrative Medicine | 3.8 | 96.2 |
| Autogenic | 2 | 98 |

**Supplementary Table 2. IOMs Utilized/Recommended on an Annual, Monthly, and Weekly Basis**

| ***Frequency - Select the integrative oncology modalities that you have utilized/recommended to manage your patients’ symptoms*** | Not Recommended/ NoResponse | | At least once annually | | At least once monthly | | At least once weekly | |
| --- | --- | --- | --- | --- | --- | --- | --- | --- |
|  | N | % | N | % | N | % | N | % |
| Acupuncture/Acupressure | 213 | 61.9 | 52 | 15.1 | 35 | 10.2 | 44 | 12.8 |
| Aromatherapy | 292 | 84.9 | 20 | 5.8 | 21 | 6.1 | 11 | 3.2 |
| Art Therapy | 282 | 82 | 32 | 9.3 | 19 | 5.5 | 11 | 3.2 |
| Autogenic | 338 | 92.3 | 4 | 1.2 | 1 | 0.3 | 1 | 0.3 |
| Ayurveda | 334 | 97.1 | 4 | 1.2 | 3 | 0.9 | 3 | 0.9 |
| Biofeedback | 324 | 94.2 | 12 | 3.5 | 7 | 2 | 1 | 0.3 |
| Biofield Therapy | 318 | 92.4 | 8 | 2.3 | 12 | 3.5 | 6 | 1.7 |
| Breathing Exercises/ Yoga | 238 | 69.2 | 16 | 4.7 | 31 | 9 | 59 | 17.2 |
| Chiropractic | 328 | 95.3 | 9 | 2.6 | 6 | 1.7 | 1 | 3 |
| Culinary Therapy | 331 | 96.2 | 1 | 3 | 4 | 1.2 | 8 | 2.3 |
| Dance Movement Therapy | 323 | 93.9 | 8 | 2.3 | 8 | 2.3 | 5 | 1.5 |
| Personalized Exercise | 240 | 69.8 | 6 | 1.7 | 25 | 7.3 | 73 | 21.2 |
| Exercise Classes | 239 | 69.5 | 7 | 2 | 33 | 9.6 | 65 | 18.9 |
| Guided Imagery | 307 | 89.2 | 8 | 2.3 | 13 | 3.8 | 16 | 4.7 |
| Health Coaching/ Supportive Counseling | 284 | 82.6 | 4 | 1.2 | 27 | 7.8 | 29 | 8.4 |
| Herbal Botanicals | 312 | 90.7 | 3 | 0.9 | 6 | 1.7 | 23 | 6.7 |
| Homeopathy | 328 | 95.3 | 2 | 0.6 | 7 | 2.0 | 7 | 2.0 |
| Hypnosis | 323 | 93.9 | 10 | 2.6 | 6 | 1.7 | 5 | 1.5 |
| Intravenous Vitamins | 335 | 97.4 | 1 | 0.3 | 3 | 0.9 | 5 | 1.5 |
| Laughter Therapy | 334 | 97.1 | 4 | 1.2 | 4 | 1.2 | 2 | 0.6 |
| Lifestyle Change Counseling and Guidance | 253 | 73.5 | 9 | 2.6 | 27 | 7.8 | 55 | 16 |
| Light Therapy | 331 | 96.2 | 5 | 1.5 | 2 | 0.6 | 6 | 1.7 |
| Manual Therapy | 323 | 93.8 | 7 | 2 | 10 | 2.9 | 4 | 1.2 |
| Massage (including Oncology Massage) | 250 | 72.7 | 20 | 5.8 | 42 | 12.2 | 32 | 9.3 |
| Medical/Nursing integrative oncology consultation | 298 | 86.6 | 6 | 1.7 | 19 | 5.5 | 21 | 6.1 |
| Meditation and mindfulness | 263 | 76.5 | 13 | 3.8 | 30 | 8.7 | 38 | 11 |
| MBSR MBCT | 289 | 84 | 10 | 2.9 | 25 | 7.3 | 20 | 5.8 |
| Music Therapy | 299 | 86.9 | 8 | 2.3 | 26 | 6.7 | 14 | 4.1 |
| Narrative Medicine | 333 | 96.8 | 2 | 0.6 | 7 | 2 | 2 | 0.6 |
| Nutrition Consultation | 237 | 68.9 | 10 | 2.9 | 41 | 11.9 | 56 | 16.3 |
| Pet Therapy | 319 | 92.7 | 6 | 1.7 | 9 | 2.6 | 10 | 2.9 |
| Peer Support | 265 | 77 | 13 | 3.8 | 39 | 11.3 | 27 | 7.8 |
| Progressive muscle relaxation | 323 | 93.9 | 4 | 1.2 | 8 | 2.3 | 9 | 2.6 |
| Qi Gong/Tai Chi | 308 | 89.5 | 6 | 1.7 | 18 | 5.2 | 12 | 3.5 |
| Reflexology | 326 | 94.8 | 5 | 1.5 | 8 | 2.3 | 5 | 1.5 |
| Resilience Training | 333 | 96.8 | 8 | 0.6 | 4 | 1.2 | 5 | 1.5 |
| Spiritual Therapy | 296 | 86 | 2 | 0.6 | 18 | 5.2 | 28 | 8.1 |
| Support Oral Vitamins | 285 | 82.8 | 2 | 0.6 | 17 | 4.9 | 40 | 11.6 |
| Traditional Healers | 325 | 94.5 | 3 | 0.9 | 8 | 2.3 | 8 | 2.3 |
| Yoga | 263 | 76.5 | 8 | 2.3 | 35 | 10.2 | 38 | 11 |
| Other | 326 | 94.8 | 1 | 0.3 | 4 | 1.2 | 13 | 3.8 |
| Other (n=1 per category): Cannabis, Dental, Health Psychology, Kampo, Molecular integrative oncology (evidence-based molecularly-targeted natural health product, supplements and re-purposed pharmaceutical) moxibustion, nature exposure, off-label drugs, orthobionomy, other allied health therapies (physio, SLP, occupational), physiotherapy, psychoanalysis, shiatsu, sweat lodge, traditional siddha medicine (Tamil literature- herbal formulations), trauma therapy, virtual reality headsets, virtual reality relaxation. |  |  |  |  |  |  |  |  |

**Supplementary Table 3. Symptom Categories and Terms Utilized (Open-Ended)**

| **Emotional** | Emotion/al symptoms, anxiety, depression, sadness, distress, psychological distress, psychological support, coping, grief, fear, fear of recurrance, burnout, loss of meaning, isolation, re-integration to life, re-integration to work, work problems, social support |
| --- | --- |
| **Pain** | Pain, neuropathy, tingling, numbness, arthralgia, muscle aches, headaches, CIPN |
| **Gastrointestinal** | Nausea, vomiting, weight loss/gain, anorexia, cachexia, muscle loss, xerostomia, mucositis, obesity, nutrition, diet, loss of appetite, malnutrition, constipation, diarrhea |
| **Fatigue** | Fatigue, weakness, malaise, sendentarism |
| **Other** | Lymphydema, arthritis, neutropenia, anemia, edema, hypotension, alopecia, immune system/support, skin issues, dental issues, trismus, nerve damage, speech abnormalities, bone necrosis/health/ostopenia, sensory alteration, self-image, metabolic disease, hearing loss |
| **Sleep** | Sleep, insomnia |
| **Hormonal** | Hormonal, hot flashes, menopausal symptoms, sexual function, sexuality, intimacy, gynecological, erectile dysfunction |
| **Mobility** | Exercise, mobility, range of motion, joint stifness, muscle stifness, falls/balance issues, physical strength, physical fitness |
| **Dyspnea** | Dyspnea, dyspnoea, breathlessness, agitation |
| **Cognitive** | Cognitive issues, cognition, brain fog |

**Supplementary Figure 1. IOMs Recommended for Active Treatment – Symptom Categories (per region)**


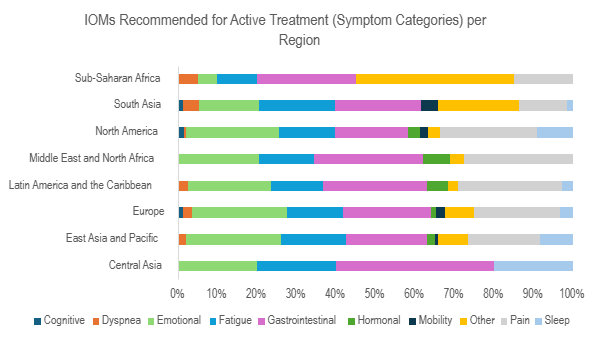


**Supplementary Figure 2. IOMs Recommended for Completed Treatment – Symptom Categories (per region)**


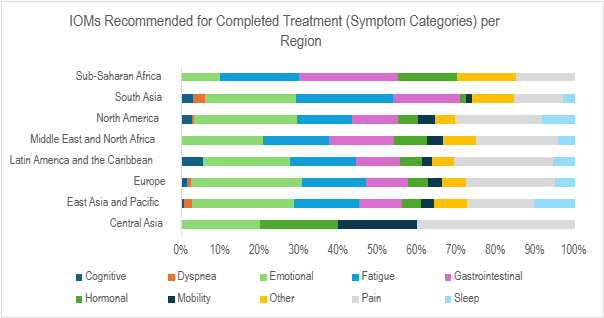

Supplement: Supplementary file 1 — Supplementary Material 1. [file 12906_2025_5157_MOESM1_ESM.docx]
