# Supplementary material for: Evaluation of integrative oncology modalities for symptom management: a MASCC/SIO global survey
Source: BMC Complement Med Ther. 2025 Nov 3;25:406. doi: 10.1186/s12906-025-05157-6 (PMC12581493; doi:10.1186/s12906-025-05157-6)
Supplement: Supplementary file 2 — Supplementary Material 2. [file 12906_2025_5157_MOESM2_ESM.pdf]

A Survey on Global Adoption of Integrative Oncology in Supportive Care

Dear Colleagues,

Due to your association with the Multinational Association of Supportive Care in Cancer (MASSC) and/or the Society of Integrative Oncology (SIO), you are invited to participate in the survey titled 'A Survey on Global Adoption of Integrative Oncology in Supportive Care'. We look forward to receiving your survey by 7/1/2023.

Our survey seeks to assess (i) the adoption of integrative oncology modalities for supportive care; (ii) your perception of patient barriers to integrative oncology for supportive care; and (iii) your perception of healthcare professionals' barriers to integrative oncology for supportive care.

- *Integrative oncology is a patient-centered, evidence-informed field of cancer care that utilizes mind and body practices, natural products, and/or lifestyle modifications from different traditions alongside conventional cancer treatments. Integrative oncology aims to optimize health, quality of life, and clinical outcomes across the cancer care continuum and to empower people to prevent cancer and become active participants before, during, and beyond cancer treatment.*

- *Supportive care is the prevention and management of the adverse effects of cancer and its treatment. This includes management of physical and psychological symptoms and side effects across the continuum of the cancer journey from diagnosis through treatment to post-treatment care.*

The survey will take between 15-20 minutes to complete. We encourage you to answer all the questions as it will provide optimal data. However, your participation in this study is voluntary, and you may withdraw from the survey at any time. Completion of the survey will allow us to utilize the data for research purposes. No identifiable information will be gathered, and the information you provide will remain completely anonymous. There is no reasonably foreseeable risk of discomfort or distress associated with your participation in this study.

It is possible to respond using many different devices, e.g. computer, tablet or mobile phones. Please complete the questionnaire in one session (there is no way to continue at a later time).

Please do not hesitate to contact Dr. Alexandre Chan (a.chan@uci.edu) or Dr. Ana Maria Lopez (anamaria.lopez@jefferson.edu) with any questions.

Looking forward to receiving your responses.

Demographics Questions

1. What is your year of birth? __________________ (*free text*)

2. What gender do you identify yourself as?
   - Male
   - Female
   - Trans-gender
   - Non-binary
   - Prefer not to answer
   - Other________

3. Which country do you primarily work/practice in? ___________ (*a drop-down list*)

4. Do you belong to an ethnic/racial minoritized group in the country/region where you practice?
   - Yes, please identify _____________(*free text*)
   - No

5. What is your current **primary** professional role?
   - Physician (working/certified in an integrative health modality):
     - Discipline: ___________
   - Physician (not working/certified in integrative health modality)
     - Specialty:_____________
   - Advance practice provider (nurse practitioner or physician assistant)
   - Dentist/Oral Surgeon
   - Psychologist
   - Nurse
   - Dental Hygienist
   - Pharmacist
   - Social Worker
   - Trainee/Student
     - Discipline:
   - Accredited exercise physiologist
   - Physiotherapist
   - Basic Science Researcher
   - Clinical Science Researcher
   - Patient advocate
   - Psycho-oncology
   - Others (please define)_: __________________ *(free text)*
   - Acupuncturist
   - Ayurvedic Medicine Practitioner
   - Fitness/Exercise Instructor
   - Health/Lifestyle Coach/Counselor
   - Homeopath
   - Massage Therapist
   - Mind-body Therapist
   - Naturopath
   - Nutritionist
   - Traditional Oriental Medicine Practitioner
   - Yoga/Qigong Instructor
   - Yoga therapist
   - Reflexologist
   - Mindfulness/meditation teacher

6. How many years have you worked in your **primary** professional role? ______ *(free text)*

7. What is your current **secondary** professional role?
   - Physician (working/certified in an integrative health modality):
     - Discipline: ____________
   - Physician (not working/certified in integrative health modality)
     - Specialty: ____________
   - Advance practice provider (nurse practitioner or physician assistant)
   - Dentist/Oral Surgeon
   - Acupuncturist
   - Ayurvedic Medicine Practitioner
   - Fitness/Exercise Instructor
   - Health/Lifestyle Coach/Counselor
   - Homeopath
   - Massage Therapist
   - Mind-body Therapist
   - Naturopath
   - Nutritionist

- Psychologist
- Nurse
- Dental Hygienist
- Pharmacist
- Social Worker
- Trainee/Student
    - Discipline:
- Accredited exercise physiologist
- Physiotherapist
- Basic Science Researcher
- Clinical Science Researcher
- Patient advocate
- Psycho-oncology
- Others (please define)__________________ *(free text)*
- Not applicable (i.e., no secondary professional role)
- Traditional Oriental Medicine Practitioner
- Yoga/Qigong Instructor
- Yoga therapist
- Reflexologist
- Mindfulness/meditation teacher

8. How many years have you worked in your **secondary** professional role? _____ *(free text)*

9. Have you received formal supportive care/palliative care training?
    - Yes, please define, fellowship/other: _____________ *(free text)*
    - No

10. Have you received formal integrative medicine training?
    - Yes, fellowship/online training program/other (please list all): ______ *(free text)*
    - No

11. Are there any formal professional education courses for integrative medicine in your country?
    - Yes
    - No
    - Not sure

12. How do you describe your primary work setting?
    - Public Sector
    - Private Sector
    - Mixed public/private
    - Academic

13. What is your annual practice work distribution?
    - Inpatient (0-100%): _________
    - Outpatient (0-100%): _________
    - Non-clinical (0-100%): _________
    - Not applicable

14. How are integrative oncology services pay for in your country (please select more than one answer):
    - Self-pay
    - Private insurance
    - Government insurance
    - Foundation/Philanthropy
    - Research only
    - Other: __________________

15. Are there medical specialists providing integrative oncology assessment / consultations in your country?
    - Yes

- No
- Unsure / Do not know

16. Are there nurses or nurse practitioners providing integrative oncology assessment / consultations in your country?
    - Yes
    - No
    - Unsure / Do not know

17. Are you a member of
    - MASCC
    - SIO
    - Both
    - None of the above

18. Select the integrative oncology modalities that you have utilized/recommended to manage your patients' supportive care issues. For those that you have selected, please provide the frequencies:

	Yes?	Frequency (only if 'yes' is selected)		
		Sometimes	Often	Always
Acupuncture / Acupressure				
Aromatherapy				
Art therapy				
Autogenic training				
Ayurveda				
Biofeedback				
Biofield therapy (Reiki, Healing Touch, Therapeutic Touch, Energy Medicine)				
Breathing exercises (include yoga-based)				
Chiropractic care				
Culinary therapies				
Dance movement therapy				
Exercise personalised assessment and program				
Exercise classes				
Guided imagery				
Health coaching /supportive counseling				
Herbal botanicals				
Homeopathy				
Hypnosis				
Intravenous vitamins				
Laughter therapy				
Lifestyle change counseliing and guidance				
Light therapy				
Manual therapy (cranial sacral, osteopathic therapy)				
Massage (including oncology massage)				
Meditation group / mindfulness practice				
Mindfulness-based stress reduction-(MBSR or MBCT) group				
Music therapy				
Narrative medicine				
Nutrition consult/assessment				
Pet therapy				
Peer support / support groups				
Progressive muscle relaxation				
Qi Gong / Tai Chi				
Reflexology				
Resilience training				
Spiritual therapy (religion, prayer)				
Supplements/oral vitamins (including medication reviews)				
Traditional healers/indigenous, specify: _________ (e.g. African, Chinese, Korean, Japanese)				
Yoga				

19. What other modalities have you utilized/recommended to patients with cancer supportive care need? _____________ *(free text)*

Supportive Care-related

20. Please provide up to 5 symptoms/supportive care issues that you would recommend integrative oncology modalities for patients undergoing active primary treatment (chemotherapy, radiation, immunotherapy)?

1. ______________________________
2. ______________________________
3. ______________________________
4. ______________________________
5. ______________________________

21. Please provide up to 5 symptoms/supportive care issues that you would recommend integrative oncology modalities for survivors who have completed primary treatment?

1. ______________________________
2. ______________________________
3. ______________________________
4. ______________________________
5. ______________________________

22. Do you think integrative oncology is underutilized in supportive care?
    - Yes, and please suggest future steps to improve the use of integrative oncology in supportive care: ______________________________ *(free text)*
    - No
    - Neutral or unsure

23. **Please rate your agreement with the following barriers regarding <u>health professionals'</u> recommendation of integrative oncology for supportive care in your practice setting:**

	Strongly disagree	Disagree	Neutral/ Unsure	Agree	Strongly Agree
Clinician-related					
Clinicians lack knowledge/understanding of integrative modalities and their potential benefits					
Clinicians lack confidence in the benefits of integrative oncology practices					
Perception that integrative oncology would delay patients' conventional treatment					
Unable to make timely referrals					
Unable to convince patients to try integrative oncology for supportive care					
Unaware of integrative oncology practice guidelines					
Clinicians worry about side effects of integrative modalities					
Integrative Modalities-related					
Lack of evidence to support use					
Lack of experience to support use					
Other supportive care services are at higher priority					
Health System-related					
Concerns regarding out-of-pocket costs to patients					
Lack of insurance coverage for integrative oncology services					
Lack of referral mechanisms/pathways					
Lack access to integrative oncology services					

24. **Please rate your agreement with the following barriers regarding <u>patient' concerns</u> with integrative oncology for supportive care in your practice setting:**

	Strongly disagree	Disagree	Neutral/ Unsure	Agree	Strongly Agree
Treatment-related					
Patients lack knowledge/understanding of integrative modalities and potential benefit					
Patients lack confidence on integrative oncology modalities					
Patients lack motivation/interest in integrative modalities					
Patients are unable to convince clinicians on wanting to try integrative modalities for supportive care					
Patients are worried to pursue these treatments					
Patients worry about side effects of integrative modalities					
Patients are too sick to receive these modalities					
Patients perceive that integrative modalities would delay conventional treatment					
Patients prefer other supportive care services					
Logistics-related					
Patients are unable to access services (transportation, distance from facility, caretaking, childcare)					
Patients lack financial support (insurance/concern regarding out of pocket expenses)					
Patients often report time/schedule conflict					
Patients complain that they are too time consuming / poor adherence to treatment schedules					

Thanks for completing this survey.
